# A fish-specific antimicrobial peptide *Ms*Piscidin2 inactivates MSRV and confers protection in largemouth bass

**DOI:** 10.3389/fimmu.2025.1629256

**Published:** 2025-06-23

**Authors:** Chenjie Fei, Ziwen Wang, Yang Hu, Li Nie, Jiong Chen

**Affiliations:** ^1^ State Key Laboratory for Quality and Safety of Agro-Products, School of Marine Sciences, Ningbo University, Ningbo, China; ^2^ Laboratory of Biochemistry and Molecular Biology, School of Marine Sciences, Ningbo University, Ningbo, China; ^3^ Key Laboratory of Aquacultural Biotechnology of Ministry of Education, Ningbo University, Ningbo, China

**Keywords:** piscidin, antimicrobial peptide, largemouth bass, *Micropterus salmoides* rhabdovirus, antiviral activity

## Abstract

Antimicrobial peptides (AMPs) represent an evolutionarily conserved component of innate immunity with broad-spectrum antimicrobial and antiviral activities. However, the antiviral potential of fish-specific piscidins against emerging aquatic viruses largely remains to be explored. In this study, we evaluated the antiviral properties of three piscidins (designated here as *Ms*Piscidin1, *Ms*Piscidin2 and *Ms*Piscidin3) identified from largemouth bass (*Micropterus salmoides*) against *Micropterus salmoides* rhabdovirus (MSRV), a major pathogen causing high mortality in farmed largemouth bass. Computational prediction and expression profiling revealed inducible expression of *Ms*Piscidins upon MSRV infection, with distinct tissue-specific patterns. Functional assays demonstrated that while *Ms*Piscidin1 and *Ms*Piscidin3 primarily modulated host antiviral responses, *Ms*Piscidin2 exhibited direct virucidal activity against MSRV. Molecular docking predicted potential interactions between *Ms*Piscidin2 and the MSRV glycoprotein, where histidine and glutamic acid residues of *Ms*Piscidin2 are positioned in close proximity to cysteine and methionine residues of the MSRV glycoprotein, supporting its capacity to directly target viral particles. *In vitro* assays further confirmed that *Ms*Piscidin2 significantly suppressed MSRV replication and attenuated cytopathic effects in a dose-dependent manner. Further, *Ms*Piscidin2 treatment conferred significant *in vivo* protection, delaying disease progression and improving survival rates in MSRV-infected juvenile bass. These findings provide the first evidence of piscidin-mediated antiviral defense against MSRV and highlight *Ms*Piscidin2 as a promising candidate for developing novel antiviral strategies in largemouth bass aquaculture.

## 1 Introduction

Antimicrobial peptides (AMPs) are evolutionarily conserved components of the innate immune system that serve as the first line of defense against a wide range of pathogens, including viruses, bacteria, fungi, and parasites (1). These small peptides, typically 10 ~ 50 amino acid in length, can be constitutively expressed in epithelia cells to provide continuous protection by suppressing microbial replication, or be rapidly induced in immune cells in response to infection (2, 3). Initially discovered for their potent bactericidal properties (4), AMPs have been extensively studied for their ability to disrupt bacterial membranes and inhibit microbial growth (5, 6). Recently, accumulating evidence suggests that AMPs also possess broad-spectrum antiviral activities (7). These peptides can interfere with viral infections through multiple mechanisms, including direct virucidal activity, inhibition of viral entry and replication, and modulation of host immune responses (8, 9).

AMPs with antiviral activity have been identified in a wide range of phylogenetically distinct species, such as mammals, birds, amphibians, and teleost fish (10). LL-37, the active form of human cathelicidin, has been reported to exert potent antiviral activities against respiratory viruses, such as influenza A virus and respiratory syncytial virus (11). Similarly, avian β-defensins have been implicated in host protection against avian influenza virus (12, 13). Further, temporins isolated from the skin of European common frog (*Rana temporaria*) were demonstrated to inhibit replications of Frog virus 3 (14). In teleost fish, hepcidins have been implicated in the suppression of nervous necrosis virus (NNV) replication in grouper species by regulating iron metabolism and immune signaling (15). Collectively, the ubiquitous presence of antiviral AMPs in various species indicated that AMP-mediated antiviral activity represents an evolutionarily conserved mechanism to enhance host resistance to viral infections.

Among fish-derived AMPs, piscidin is a unique family only present in teleost fish (16). Structurally, these small peptides are initially produced as a prepropeptide consisting of ~64 to 89 amino acids, which undergoes proteolytic cleavage to remove the N-terminal signal peptide and the C-terminal prodomain prior to the release of the mature peptide of 18 to 26 amino acids in length (17). To date, a number of small peptides, such as piscidin, epinecidin and pleurocidin, belong to the piscidin family has been identified, and functional characterizations further demonstrated their potent antiviral activities. For example, epinecidin derived from orange-spotted grouper (*Epinephelus coioides*) effectively inhibited NNV and foot-and-mouth disease virus via distinct modes of action (15, 18). This broad-spectrum antiviral activity further underscores its promise as an alternative candidate against viral infections.

Largemouth bass (*Micropterus salmoides*) is one of the most economically important freshwater species in aquaculture. However, the expansion of largemouth bass farming has been accompanied by a rise in infectious diseases, which threaten the sustainability of the industry. Among these, *Micropterus salmoides* rhabdovirus (MSRV) represents a major threat and has caused severe hemorrhagic disease outbreaks associated with high mortality rates in farmed juvenile fish, primarily due to its ability to establish systemic infection with pathological lesions detected in multiple tissues, including intestine, liver, muscle, brain and spleen (19–21). Despite efforts to develop vaccines and chemical treatments, current control measures remain inadequate, necessitating the search for alternative antiviral strategies. Three largemouth bass piscidins (*Ms*Piscidin), designated as *Ms*Piscidin1, *Ms*Piscidin2 and *Ms*Piscidin3 here, have been identified in a previous study and functional characterizations demonstrated potent bactericidal activity against multiple aquatic pathogens (22). Although the antibacterial properties of *Ms*Piscidin are well characterized, their involvement in antiviral defense remains largely unexplored. A detailed investigation into their activity against MSRV infection will broaden our understanding of teleost-specific AMPs, extending their functional relevance beyond antibacterial action.

In the present study, the antiviral potential of *Ms*Piscidins was first predicted in silico and validated by temporal expression analysis following MSRV infection. Functional assays revealed that *Ms*Piscidin1 and *Ms*Piscidin3 modulated host immune responses, while *Ms*Piscidin2 directly suppressed MSRV replication in a dose-dependent manner and reduced cytopathic effects (CPE) *in vitro*. Further *in vivo* assay demonstrated that *Ms*Piscidin2 significantly improved survival in infected juvenile largemouth bass. Taken together, this represents the first report on antiviral activities of *Ms*Piscidins against MSRV infection and highlight their potential as novel antiviral agents in aquaculture.

## 2 Materials and methods

### 2.1 Cell culture and fish rearing

Epithelioma Papulosum Cyprini (EPC) cells were kindly provided by Dr. Yi-bing Zeng in Yangtze River Fisheries Research Institute (Wuhan, China) and cultured as previously described (23). Briefly, cells were grown in medium 199 (M199; HyClone, USA) supplemented with 10% fetal bovine serum (FBS; SIJIQING, China), penicillin (100 IU/mL; Thermo fisher scientific, USA) and streptomycin (100 μg/mL; Thermo fisher scientific, USA) at 25 °C and 5% CO_2_.

Juvenile largemouth bass were purchased from the Yangyang fishery breeding company in Guangdong, China and all fish were maintained at 28 ± 0.5°C in a flow-through water system on a simulated natural photoperiod. Fish were acclimated to this environment for at least two weeks prior to any experiment. All experiments involving animals were approved by the Ningbo University Institutional Animal Care and Use Committee and were carried out in compliance with the National Institutes of Health’s Guide for the Care and Use of Laboratory Animals.

### 2.2 Viral propagations

*Micropterus salmoides* rhabdovirus (MSRV) strain (MSRV-YH01) was kindly provided by Dr. Jia-yun Yao in Zhejiang Institute of Freshwater Fisheries (Huzhou, China) and propagated in EPC cells as previously described (24, 25). Briefly, supernatants containing viral particles originally stored at -80°C were thawed. Prior to the viral infection, concentration of FBS in the culturing medium was reduced to 2% and then 100 μL of aforementioned viral supernatants were added to EPC cells (1×10^5^ cells). After 72 h, the supernatant was collected, aliquoted and stored at -80°C. The tissue culture infective dose (TCID_50_) was determined using Reed-Muench method (26).

### 2.3 Synthetic peptides

Three largemouth bass piscidins, i.e., *Ms*Piscidin1(GenBank: MT681907), *Ms*Piscidin2 (GenBank: MT681908) and *Ms*Piscidin3 (GenBank: MT681909) were identified and their structures were analyzed in a previous study (22). In this study, mature peptides of three *Ms*Piscidins were provided by GL Biochem Ltd. (Shanghai, China). The synthesized *Ms*Piscidins were purified by RP-HPLC and the purity was >95%.

### 2.4 Characterization of physiochemical properties and prediction of antiviral potentials of *Ms*Piscidins

Physicochemical characteristics, i.e., isoelectric point (pI) and charge of *Ms*Piscidins were calculated using PepDraw (https://pepdraw.com). Antiviral potentials of *Ms*Piscidins were predicted using AI4AVP (http://axp.iis.sinica.edu.tw/AI4AVP/).

### 2.5 RT-qPCR analysis of *Ms*Piscidins expression in tissues of infected largemouth bass

To profile the expression of *Ms*Piscidins in response to the viral infection, largemouth bass were infected by intraperitoneal injection of MSRV containing supernatants at 5×10^2^ TCID_50_ or virus negative supernatants alone as controls. After 24 h infection, selected immune-relevant tissues, including liver, spleen, intestine, skin, and gills were collected for total RNA extraction, reverse transcription and RT-qPCR analysis as previously described (27). Briefly, Total RNA was extracted using TriQuick Reagent (Solarbio, China) and reverse transcribed using HiScript^®^ III All-in-one RT SuperMix Perfect for qPCR (Vazyme, China) following the manufacturers’ instructions. Obtained cDNAs were then analyzed for *Ms*Piscidins expression by RT-qPCR using Taq Pro Universal SYBR qPCR Master Mix (Vazyme, China). Then the RT-qPCR analysis was performed on an ABI QuantStudio 3 real-time PCR System (Applied Biosystems, USA) using primers listed in **Supplementary Table S1**. Thermocycling parameters were as follows: 95 °C for 30 s, followed by 40 cycles of 10 s at 95 °C and 30 s at 60 °C. The relative gene expression of *Ms*Piscidins was calculated using the 2^-ΔΔCT^ method normalized to the endogenous control gene (i.e., *β-actin*).

### 2.6 Cytotoxicity assay

To evaluate cytotoxic effects and determine safe dose of *Ms*Piscidins, synthesized peptides used in this study were serially diluted and then added to EPC cells (~90% confluency in 96-well plates). After 24h incubation, cell viability was measured using CCK-8 assay following the manufacturers’ instructions (Beyotime, China). The optical density (OD) of each sample at 450nm was measured using a microplate reader. Percentage of cell viability was calculated using the following equation:(OD _experimental group_ – OD _blank group_)/(OD _control group_ – OD _blank group_) × 100%. EPC cells treated with PBS were used as the control group, while wells contain only reaction solution were used as the blank group. Cell viability > 90% after *Ms*Piscidins incubation is considered as non-toxic.

### 2.7 Molecular docking

The tertiary structures of the MSRV glycoprotein (MSRV G protein; accession number: QBF51718.1) and *Ms*Piscidin2 were predicted using AlphaFold3 (https://alphafoldserver.com/). The predicted structures were evaluated based on the per-residue confidence score, and the highest-ranked models were selected and protein data bank (PDB) files were downloaded for molecular docking analysis using HADDOCK2.4 (28, 29). Specifically, the resulting PDB files of predicted MSRV G protein and MsPiscidin2 structures were uploaded to the HADDOCK web server (https://rascar.science.uu.nl/haddock2.4/), and default docking parameters were applied. The top-ranked cluster with the lowest HADDOCK score was selected and visualized using PyMOL (version 2.1, Schrödinger, LLC). Briefly, MSRV G protein-*Ms*Piscidin2 complex generated from HADDOCK2.4 was rendered in cartoon representation, with critical contact points highlighted using stick models.

### 2.8 Functional characterization of antiviral activities of *Ms*Piscidins

To screen the antiviral potential of *Ms*Piscidins, a viral dose of 1×10^3^ TCID_50_ MSRV was pre-incubated with *Ms*Piscidin1 (6.25 μg/mL), *Ms*Piscidin2 (6.25 μg/mL), *Ms*Piscidin3 (6.25 μg/mL) or equal volume of serum-free M199 for 2 h at room temperature. EPC cells were then exposed to the pre-treated MSRV for 2 h, followed by three washes with PBS and cultured as aforementioned with the exception that the FBS concentration in the culturing medium was reduced to 2%. Alternatively, EPC cells were pre-incubated with *Ms*Piscidin1, *Ms*Piscidin2 or *Ms*Piscidin3 at the concentration of 6.25 μg/mL for 12 h, followed by three washes with PBS, then infected and cultured as described above. After 48 h, EPC cells and supernatants were collected for total RNA extraction, reverse transcription and RT-qPCR analysis as described above to assess the expression of viral G gene using the primers listed in **Supplementary Table S1**.

To confirm the direct antiviral capability of *Ms*Piscidin2, a viral dose of 1×10^3^ TCID_50_ MSRV was pre-incubated with serially diluted *Ms*Piscidin2 (i.e., 100 μg/mL, 50 μg/mL, 25 μg/mL, 12.5 μg/mL, 6.25 μg/mL and 3.125 μg/mL) before infecting EPC cells as detailed above to assess the CPE using light microscope and expression of viral G gene.

To further investigate the temporal effects of *Ms*Piscidin2 treatment on the viral replication of infected cells, a viral dose of 1×10^3^ TCID_50_ MSRV was pre-incubated with *Ms*Piscidin2 (i.e., 6.25 μg/mL) and EPC cells were infected as detailed above. Cells and supernatants were collected at 24 h, 48 h and 72 h for evaluating the expression of viral G gene using RT-qPCR as detailed above.

### 2.9 *In vivo* analysis of *Ms*Piscidin2 against MSRV infection

To determine the *in vivo* toxicity of *Ms*Piscidin2, largemouth bass (n=20 per group) were intraperitoneally injected 40 μL PBS, or 40 μL *Ms*Piscidin2 of different concentrations (i.e., 0.1, 1 and 10 mg/kg). The survival was monitored for 15 days and plotted accordingly. To further investigate the protective effect of *Ms*Piscidin2 on MSRV infection *in vivo*, ninety juvenile largemouth bass were randomly selected and distributed into three groups and intraperitoneally injected with 20 μL PBS, 20 μL MSRV (5×10^2^ TCID_50_) and 20 μL MSRV (5×10^2^ TCID_50_) combined with the same volume of *Ms*Piscidin2 (1 mg/kg), respectively. In the next 15 days, the number of live fish was monitored every day to plot the survival curve.

## 3 Results

### 3.1 MSRV infection induces tissue-specific upregulation of *MsPiscidin* genes in largemouth bass

**Figure 1:**
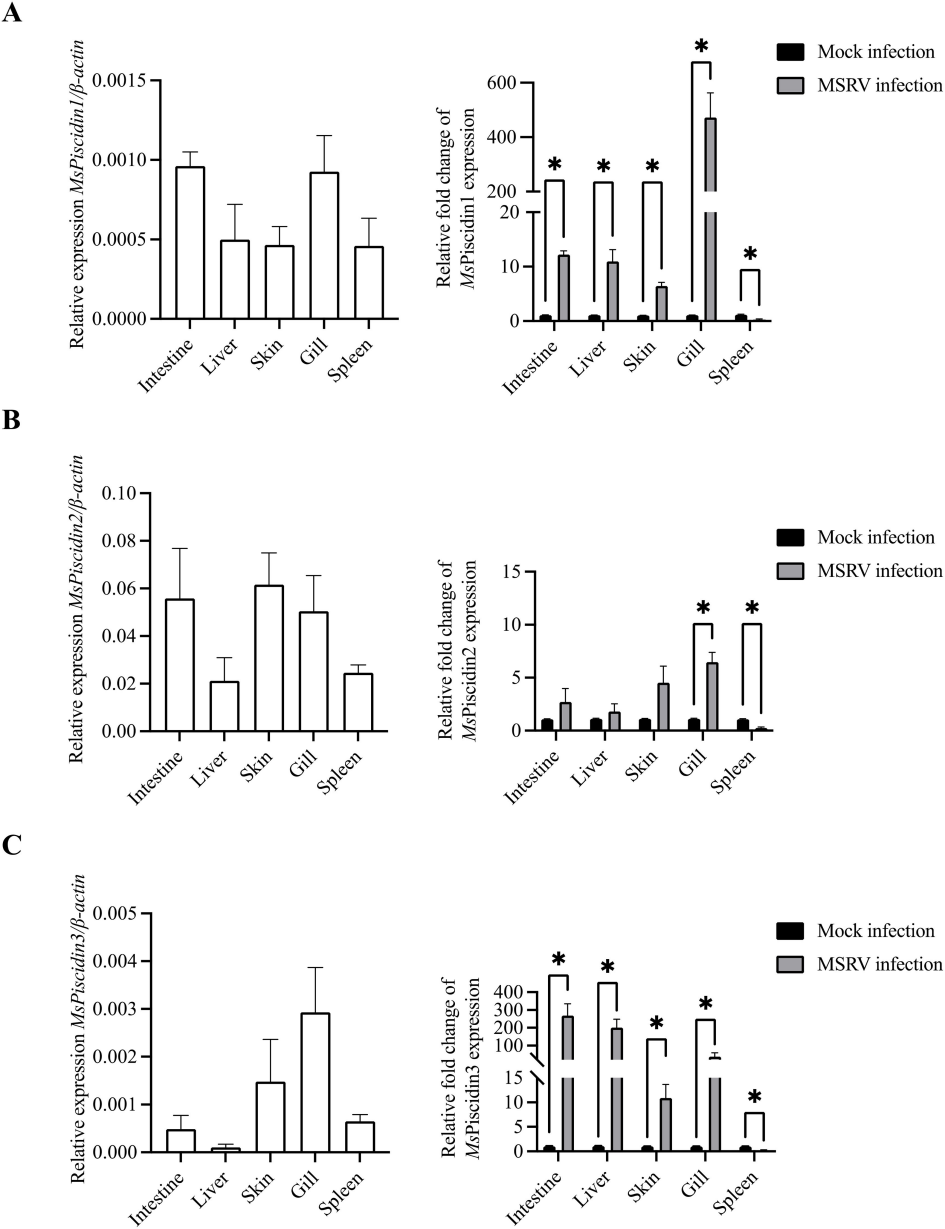
Tissue-specific expression profiles of *Ms*Piscidins in largemouth bass under basal and MSRV-infected conditions. qRT-PCR analysis of mRNA expression levels of *Ms*Piscidin1 **(A)**, *Ms*Piscidin2 **(B)**, and *Ms*Piscidin3 **(C)** under the basal condition or following MSRV infection. The left panels show the basal expression levels of each gene in healthy fish, normalized to the endogenous β-actin. The right panels show relative expression of *Ms*Piscidin transcript levels following MSRV infection compared to the mock infection group using 2^-△△Ct^ method. Data represent the mean ± SEM of three biological replicates (n = 3). Asterisks indicate significant differences compared to the control group (*p < 0.05).

To investigate the innate immune response of largemouth bass to MSRV infection, RT-qPCR analysis was performed to evaluate expression profiles of three *MsPiscidin* genes in selected tissues after 48 h infection. Specifically, *MsPiscidin1* and *MsPiscidin3* exhibited similar tissue-specific expression profiles that significant up-regulations were observed in all examined tissues except spleen following MSRV infection, though with differing magnitudes (**Figures 1A, C**). The highest expression was found in the gill (~470-fold) and intestine (~270-fold) for *MsPiscidin1* and *MsPiscidin3*, respectively. Marked increases were also observed in liver and skin tissues. Of note, a significant reduction in *MsPiscidin* genes expression was found in spleen. In contrast, *Mspiscidin2* expression was only up-regulated ~5-fold in gill, whereas expression in the intestine, liver, and skin slightly increase but not significantly different compared to the control; similarly, a significant reduction of gene expression level was also obvious in the spleen (**Figure 1B**). Further computational predictions revealed potential antiviral potentials of *Ms*Piscidins, with antiviral probabilities of 0.983, 0.995 and 0.919 for *Ms*Piscidin1, *Ms*Piscidin2 and *Ms*Piscidin3, respectively (**Table 1**).

**Table 1:** Physicochemical characteristics and antiviral potentials of the *Ms*Piscidins.

Name	Mature peptide sequence	Net charge	Isoelectric point	Antiviral probability^*^
*Ms*Piscidin1	FLGTLLHGAVHVSKILHGIMGGDH	0	7.98	0.983
*Ms*Piscidin2	FLKHIKSFWRGAKAIFRGARQGWREHR	+7	12.67	0.995
*Ms*Piscidin3	FIFHVIKGLFHAGKMIHGLVTRRRH	+5	12.79	0.919

*Antiviral probability of selected peptides was predicted using AI4AVP program.

### 3.2 *Ms*Piscidins exhibit dose-dependent cytotoxicity in EPC cells

**Figure 2:**
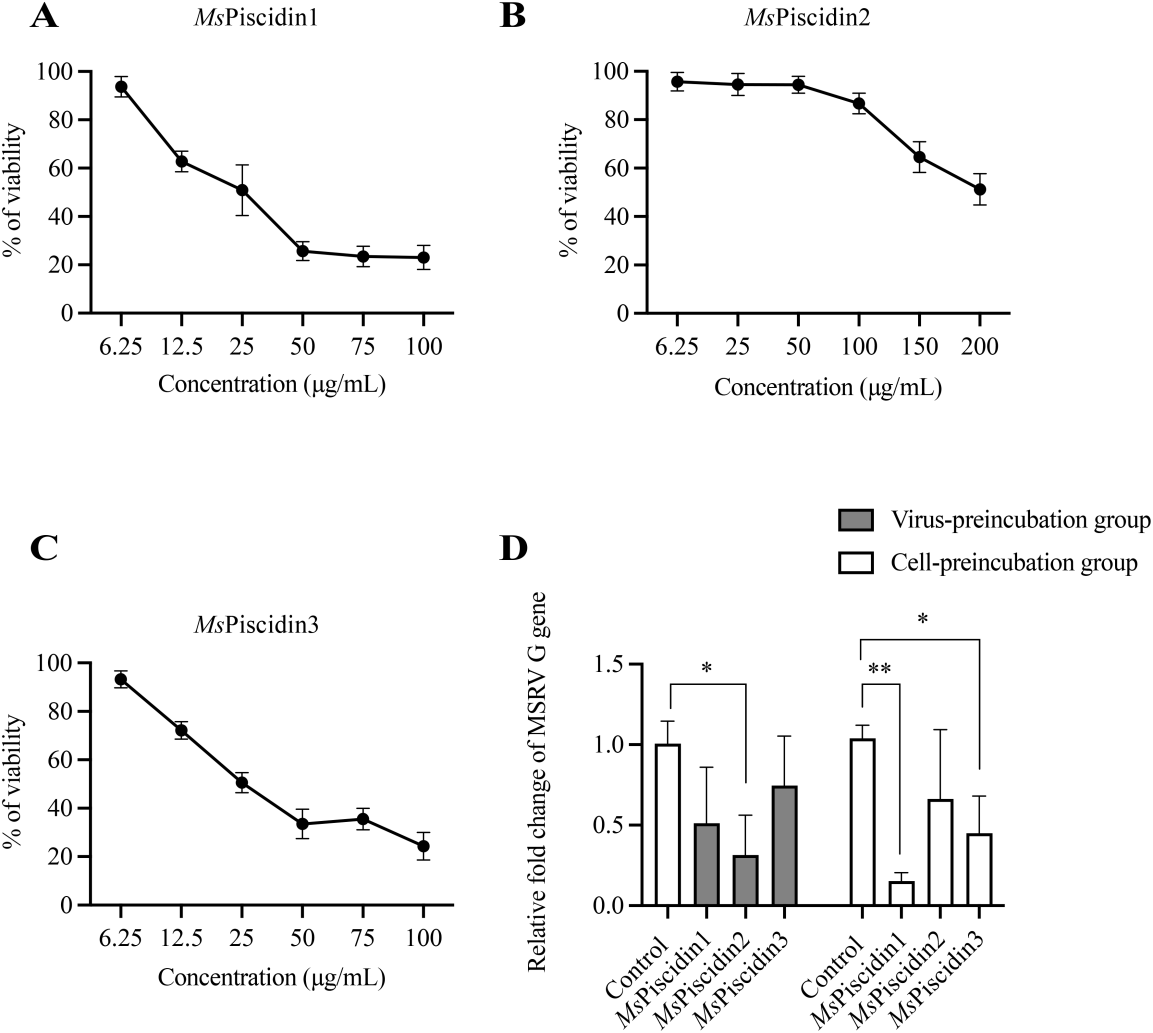
Cytotoxicity and antiviral activity of *Ms*Piscidins in EPC cells. EPC cells were incubated with indicated concentrations of *Ms*Piscidin1 **(A)**, *Ms*Piscidin2 **(B)**, or *Ms*Piscidin3 **(C)** and after 48 h incubation, cell viability was assessed using the CCK-8 assay. Data are expressed as mean ± SEM of three independent experiment, each performed in triplicates. **(D)** In the virus-preincubation group (grey bar), 1×10^3^ TCID_50_ MSRV virus were pre-incubated with PBS (i.e., control group) or respective *Ms*Piscidins at the concentration of 6.25 μg/mL. After 2 h incubation, pre-incubated MSRV viruses were further used to infect EPC cells for 2h. In the cell-preincubation group (white bar), EPC cells were pre-treated with PBS or respective *Ms*Piscidins at the concentration of 6.25 μg/mL. After 12 h treatment, EPC cells were further infected with 1×10^3^ TCID_50_ MSRV for 2h. EPC cells were collected after 48 h and the relative expression levels of MSRV G gene were calculated using 2^-△△Ct^ method and relative to the control group. Data represent the mean ± SEM of three biological replicates (n = 3). Asterisks indicate significant differences compared to the control group (*p < 0.05, **p < 0.01).

To evaluate the cytotoxic potential of synthetic *Ms*Piscidin peptides, EPC cells were treated with increasing concentrations of *Ms*Piscidin1, *Ms*Piscidin2, or *Ms*Piscidin3 and after 24 h, CCK-8 assay was performed to assess cell viability. Specifically, *Ms*Piscidin1 and *Ms*Piscidin3 demonstrated a similar dose-dependent cytotoxic effect on EPC cells. Both synthetic peptides impaired cell survival at 12.5 µg/mL, with viability dropping below 50% at concentrations ≥50 µg/mL (**Figures 2A, C**). In contrast, *Ms*Piscidin2 exhibited a markedly different cytotoxic profile that cell viability remained above 90% up to 50 µg/mL. However, at higher concentrations (i.e., 100, 150 and 200 µg/mL), percentage of viable cells was reduced to ~86%, ~64% and ~51%, respectively (**Figure 2B**).

### 3.3 *Ms*Piscidins inhibit MSRV replication via distinct mechanisms

To assess the antiviral activity of *Ms*Piscidin peptides against MSRV, EPC cells or MSRV were pre-incubated with *Ms*Piscidin peptides, followed by RT-qPCR analysis of MSRV G gene expression to examine infectivity of pre-treated MSRV and viral resistance of pre-treated EPC cells. As shown in **Figure 2D**, pre-incubating MSRV only with *Ms*Piscidin2 significantly reduced viral gene expression compared to the control, indicating that *Ms*Piscidin2 capable of inactivating MSRV directly. In comparison, a significant reduction in MSRV G gene expression was only seen when EPC cells were pre-treated with *Ms*Piscidin1 or *Ms*Piscidin3, suggesting that both peptides indirectly exert antiviral effects, likely through modulation of host cell resistance to viral infection.

### 3.4 *Ms*Piscidin2 directly inactivates MSRV replication *in vitro*

**Figure 3:**
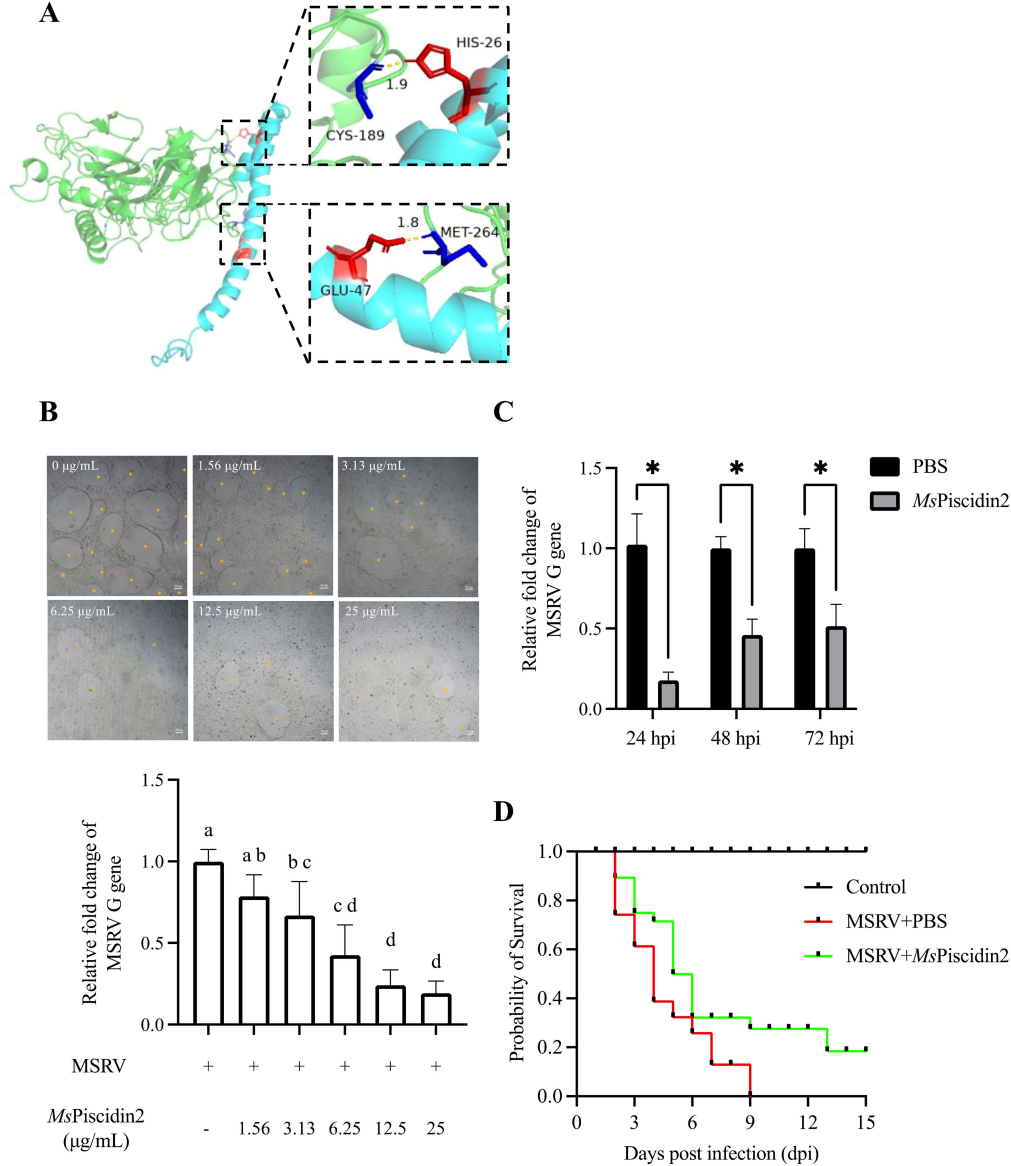
Mechanistic and functional analysis of *Ms*Piscidin2 *in vitro* and *in vivo* activity against MSRV infection. **(A)** Molecular docking analysis illustrating the interaction between the MSRV G protein (green) and the *Ms*piscidin2 (cyan). Predicted binding interfaces are highlighted with dashed boxes, showing key interacting residues in red and blue. In the upper inset, CYS-189 (blue) of MSRV G protein is positioned near HIS-26 (red) of *Ms*piscidin2 with an interatomic distance of 1.9 Å, suggesting potential hydrogen bonding or van der Waals interaction. In the lower inset, GLU-47 (red) of *Ms*piscidin2 is in close proximity (1.8 Å) to MET-264 (blue) of MSRV G protein, indicating another potential interaction hotspot. Distances are given in angstroms (Å). **(B)** EPC cells were infected with 1×10^3^ TCID_50_ MSRV pre-treated with indicated concentrations of *Ms*Piscidin2 or PBS as a positive control. After 48 hours, cells were imaged using light microscope and the cytopathic effect was indicated by yellow asterisks (top pane), followed by the qRT-PCR analysis of the relative mRNA expression of the MSRV G gene (bottom histogram). The relative expression levels were calculated using 2^-△△Ct^ method and relative to the positive control group. Data represent the mean ± SEM of three biological replicates (n = 3). Different letters indicate significantly different between groups (p < 0.05). **(C)** EPC cells were infected with MSRV virus pre-incubated with either *Ms*Piscidin2 or PBS (control group) for 2 hours. After infection, the medium was replaced with 2% FBS-containing medium. Cells and supernatants were harvested at indicated time points to quantify MSRV G gene expression using qRT-PCR. Gene expression levels are shown as relative fold changes normalized to respective control groups. Data are shown as mean ± SEM of three biological replicates (n = 3). Asterisks indicate statistically significant differences between *Ms*Piscidin2 and respective control groups (*p* < 0.05). **(D)** Survival curves of largemouth bass following MSRV infection. Ninety juvenile fish were randomly divided into three groups, i.e., control group that fish were intraperitoneally injected with PBS (black line), infection group that fish were intraperitoneally injected with 5×10^2^ TCID_50_ MSRV (red line), and treatment group that fish received intraperitoneal injection of 5×10^2^ TCID_50_ MSRV co-administrated with *Ms*Piscidin2 (green line). Fish were then monitored over 15 days and number of surviving individuals was recorded and plotted accordingly.

Molecular docking analysis was firstly performed to assess potential interactions between *Ms*Piscidin2 and MSRV, which might account for the direct antiviral activities of this peptide. Indeed, two residues in *Ms*Piscidin2 (i.e., histidine at position 26 and glutamic acid at position 47) were identified as likely interacting with cysteine and methionine residues in MSRV G protein (**Figure 3A**). To further confirm the direct viral inactivation of *Ms*Piscidin2, RT-qPCR analysis was performed to investigate the infectivity of MSRV after pre-incubation with increasing concentration of *Ms*Piscidin2. The result shown that *Ms*Piscidin2 significantly suppressed the replication of MSRV in a dose-dependent manner; the highest concentration (i.e., 25 μg/mL) tested reduced the relative expression of viral G gene to ~ 20% compared to the control group (**Figure 3B**). Consistently, CPE was also attenuated in cells infected with pre-treated MSRV in a manner proportional to *Ms*Piscidin2 concentrations (**Figure 3B**).

To further investigate the temporal dynamics of *Ms*Piscidin2 antiviral effect, MSRV was pre-incubated with *Ms*Piscidin2 prior to infection. RT-qPCR analysis demonstrated that viral replication was significantly suppressed at 24, 48, and 72 hours post infection (hpi), with maximum reduction to ~17% at 24 hpi (**Figure 3C**).

### 3.5 *Ms*Piscidin2 protects largemouth bass from MSRV infection *in vivo*

To determine the *in vivo* toxicity of *Ms*Piscidin2 and define a safe concentration range, largemouth bass were intraperitoneally injected with increasing doses of *Ms*Piscidin2 (0.1, 1, and 10 mg/kg), and results shown no mortality was observed in the control group (PBS injection) and in groups treated with 0.1 mg/kg or 1 mg/kg *Ms*Piscidin2, indicating that these doses were tolerated; in contrast, deceased fish were observed at day 2 and day 3 in the group receiving the highest dose (**Supplementary Figure S1**). To further evaluate the *in vivo* protective efficacy of *Ms*Piscidin2 against MSRV infection, survival analysis was performed and MSRV-infected fish exhibited rapid mortality, with survival rate dropping below 50% by 4 days post infection (dpi) and all fish died by 9 dpi. In comparison, survival rate of infected fish co-administrated with *Ms*Piscidin2 was pronouncedly increased, with delayed onset of mortality and a final survival rate of ~20% (**Figure 3D**).

## 4 Discussion

AMPs represent a critical component of innate immunity in teleost fish and contribute to defending against a range of incoming infectious agents (30). To date, a number of AMPs identified in teleost has been demonstrated potent antiviral activities against aquatic viruses, such as Singapore grouper iridovirus (SGIV) and NNV (31, 32). However, antiviral potentials of piscidin, the fish-specific AMPs, remain largely unknown. In this study, we provide the first evidence that *Ms*Piscidin2 exerts potent antiviral activity against MSRV. Using computational prediction, expression profiling, *in vitro* functional assays and *in vivo* infection models, we demonstrate that *Ms*Piscidin2 directly inactivates MSRV particles, significantly suppresses viral replication along with reduced CPE, and confers partial protection in infected juvenile fish. These findings highlight *Ms*Piscidin2 as a promising candidate for antiviral therapy in aquaculture and provide new insights into the antiviral capacity of fish-specific AMPs.

The computational prediction program used in this study (i.e., AI4AVP) infers antiviral potential by analyzing peptide sequence features, such as net charge, hydrophobicity, and structural motifs, through machine learning models trained on large, curated datasets of experimentally validated antiviral peptides (33). Importantly, the relatively short length and well-defined physicochemical properties of AMP make them especially amenable to computational modeling, allowing for more precise identification of functional motifs and prediction of bioactivity. Indeed, this in silico approach has been widely used as an initial step to prioritize AMP candidates with high antiviral potential for subsequent experimental validation.

The observed tissue-specific upregulation of *Ms*Piscidins upon MSRV infection suggests their likely role in anti-viral immunity. *Ms*Piscidin1 and *Ms*Piscidin3 were robustly induced in key barrier tissues, including the gills, intestine and skin, consistent with expression profiles of piscidins observed in other teleost species when challenged with bacteria and viral mimics (34, 35). Interestingly, *Ms*Piscidin2 expression was only significantly induced in gills and remained largely unchanged in other tissues, indicating that its antiviral role may not rely on pathogen-induced upregulation but rather on constitutive expression, of which is sufficient for antiviral effects.

Functional assays revealed distinct antiviral mechanisms among the *Ms*Piscidins. Specifically, *Ms*Piscidin1 and *Ms*Piscidin3 indirectly suppressed viral replication, likely by priming host cells through up-regulating immune-relevant genes (e.g., interferons and interferon-stimulated genes) as seen in other immunomodulatory AMPs (36–38). This immunomodulatory effect can enhance the basal antiviral state of the cells, rendering them more resistant to subsequent MSRV infection. In comparison, *Ms*Piscidin2 directly inactivated viral particles. The polycationic nature of *Ms*Piscidin2 may account for this discrepancy in the mode of action and mechanistically, *Ms*Piscidin2 could directly inserted into the outer membrane of MSRV via electrostatic interactions as enveloped viruses are normally negatively charged (39). Similar membrane-disrupting properties have been observed in other AMPs, such as LL-37 and temporins, which target enveloped viruses (40, 41). Consistently, hepcidin and epinecidin identified in Nile tilapia (*Oreochromis niloticus*) can cause aggregation of viral particles after incubation, likely disrupting the viral membrane (15, 42). Although the potential interactions between *Ms*Piscidin2 and MSRV G protein were shown using molecular docking analysis and two residues within the *Ms*Piscidin2 were identified, further mutagenesis analysis is required to validate the contribution of these two residues to the antiviral activity of *Ms*Piscidin2. The direct anti-viral activity of *Ms*Piscidin2 was further confirmed via pre-incubation of MSRV with increasing concentrations of *Ms*Piscidin2, of which led to a significant and dose-dependent reduction in the viral replication and CPE. Consistently, temporal analysis of viral replication demonstrated significant inhibitions of viral replication at all tested time points and further confirmed that *Ms*Piscidin2 exhibited sustained inhibitory effects on MSRV replication.

The *in vivo* protective efficacy of *Ms*Piscidin2 treatment further supports its antiviral potentials against MSRV infection. Co-administration of *Ms*Piscidin2 with MSRV significantly delayed disease progression and increased survival rates in infected juvenile largemouth bass. Although *Ms*Piscidin1 and *Ms*Piscidin3 also demonstrated promising *in vitro* activities in enhancing cellular resistance to MSRV infection, their *in vivo* protective effects are not examined in this study due to their immunomodulatory nature. As their antiviral efficacy may depend on complex host immune dynamics that vary with the timing of peptide administration and viral infection, making it more challenging to interpret their *in vivo* antiviral activities using the current infection model. However, the encouraging *in vitro* data warrants further investigation and their *in vivo* protective effects will be explored when more mechanistic details regarding to their *in vitro* antiviral activities are obtained. In contrast to the potent *in vitro* antiviral efficacy of *Ms*Piscidins2, the survival rate of infected fish following *Ms*Piscidins2 is not optimal. This observed discrepancy may derive from the differing nature of *in vitro* and *in vivo* systems; *in vitro* models offer a controlled environment where the *Ms*Piscidins2 concentration and exposure duration to viral particles are precisely maintained, whereas *in vivo* conditions involve rapid degradation of peptides by endogenous proteases, different clearance rates and variable distribution sites, all of which can reduce antiviral efficacy of *Ms*Piscidins2 *in vivo*. Consequently, the practical application of AMPs in combating viral infection in aquaculture is still limited and further engineering of *Ms*Piscidins2 (e.g., peptide cyclization) to increase its stability and optimization of delivery system to prolong the presence of administrated peptides are required.

In conclusion, our study identifies *Ms*Piscidin2 as a potent antiviral peptide capable of directly inactivating MSRV, reducing viral replication *in vitro*, and enhancing fish survival *in vivo*. These findings expand our knowledge of antiviral functions of piscidins and establish a foundation for the development of AMP-based interventions against viral pathogens in aquaculture.

## Data Availability

The raw data supporting the conclusions of this article will be made available by the authors, without undue reservation.
