# Supplementary material for: A fish-specific antimicrobial peptide MsPiscidin2 inactivates MSRV and confers protection in largemouth bass
Source: Front Immunol. 2025 Jun 23;16:1629256. doi: 10.3389/fimmu.2025.1629256 (PMC12229855; doi:10.3389/fimmu.2025.1629256)
Supplement: Supplementary file 1 [file Table1.docx]

Supplementary Material

# Supplementary table 1 Sequences of primer pairs used in the present study

| Genes |  | Primer sequences (from 5′ to 3′) |
| --- | --- | --- |
| *β-actin* | Forward | *GCTATGTGGCTCTTGACTTCGA* |
|  | Reverse | *CCGTCAGGCAGCTCATAGCT* |
| *MSRV G* | Forward | AAGAGCCCGAGAGAAAAAT |
|  | Reverse | TGAATAGCGGTCCATCAAC |
| *MsPiscidin-1* | Forward | TATTGTGATCTTTCTCGTGTTGTCC |
|  | Reverse | CTGCTCTTGGACACCGTGGT |
| *MsPiscidin-2* | Forward | GATCTTTCTGGTGCTGTCGCT |
|  | Reverse | GCTGCTTAGACAAGGCTCTGTG |
| *MsPiscidin-3* | Forward | GTGCATCACCCTCTTTCTTGTG |
|  | Reverse | TTCCATGCCATGTCGTCTCC |

**Supplementary figure 1.** Determination of non-toxic *in vivo* concentrations of *Ms*Piscidin2. To characterize the *in vivo* toxicity profile of *Ms*Piscidin2 and determine a non-lethal concentration, largemouth bass (n=20 per group) were intraperitoneally injected with PBS, or *Ms*Piscidin2 at 0.1 mg/kg (red line), 1 mg/kg (green line), or 10 mg/kg (blue line). Survival rate was monitored daily for 15 days and the number of surviving individuals was recorded and plotted accordingly.
